# Intramyocardial abscess and endoventricular thrombosis: a complex case

**DOI:** 10.1007/s10554-024-03088-w

**Published:** 2024-04-13

**Authors:** Luca Rodella, Davide Pegorin, Roberta Rosati, Michela Raffo, Enrico Vizzardi, Marco Metra

**Affiliations:** Department of Medical-Surgical Specialty, Radiology and Health Care, Cardiovascular Disease Section, School of Medicine, Spedali Civili Hospital, Via Naviglio Grande 74, Brescia, 25123 Italy

**Keywords:** Abscess, Antibiotic therapy, Infective endocarditis, Echocardiogram

## Abstract

Infective endocarditis (IE) is today a public health problem, as the recent ESC Guidelines have also recalled. Abscesses can be complications of IE and their presence means that the infection is not controlled. We describe the complex case of a 57-year-old patient, presented in ED for fever and oleocranical bursitis, increase of cardiac enymes at blood samples. He was admitted to our Cardiology Unit because TTE showed a floating peduncolated formation in the left ventricle. The susequent TEE documented also the presence of a myocardial abscess, confirmed at cardiac MRI. Blood cultures were positive for MSSA and the man received specific antibiotic therapy. Anticoagulation treatment was started with UFH and then switched to Warfarin, surgical approach of the lesion would have been too dangerous according to Cardiac Surgeons. Serious and sudden neurological complications then followed, leading the patient to brain death in ICU.

## Case report

**Fig. 1:**
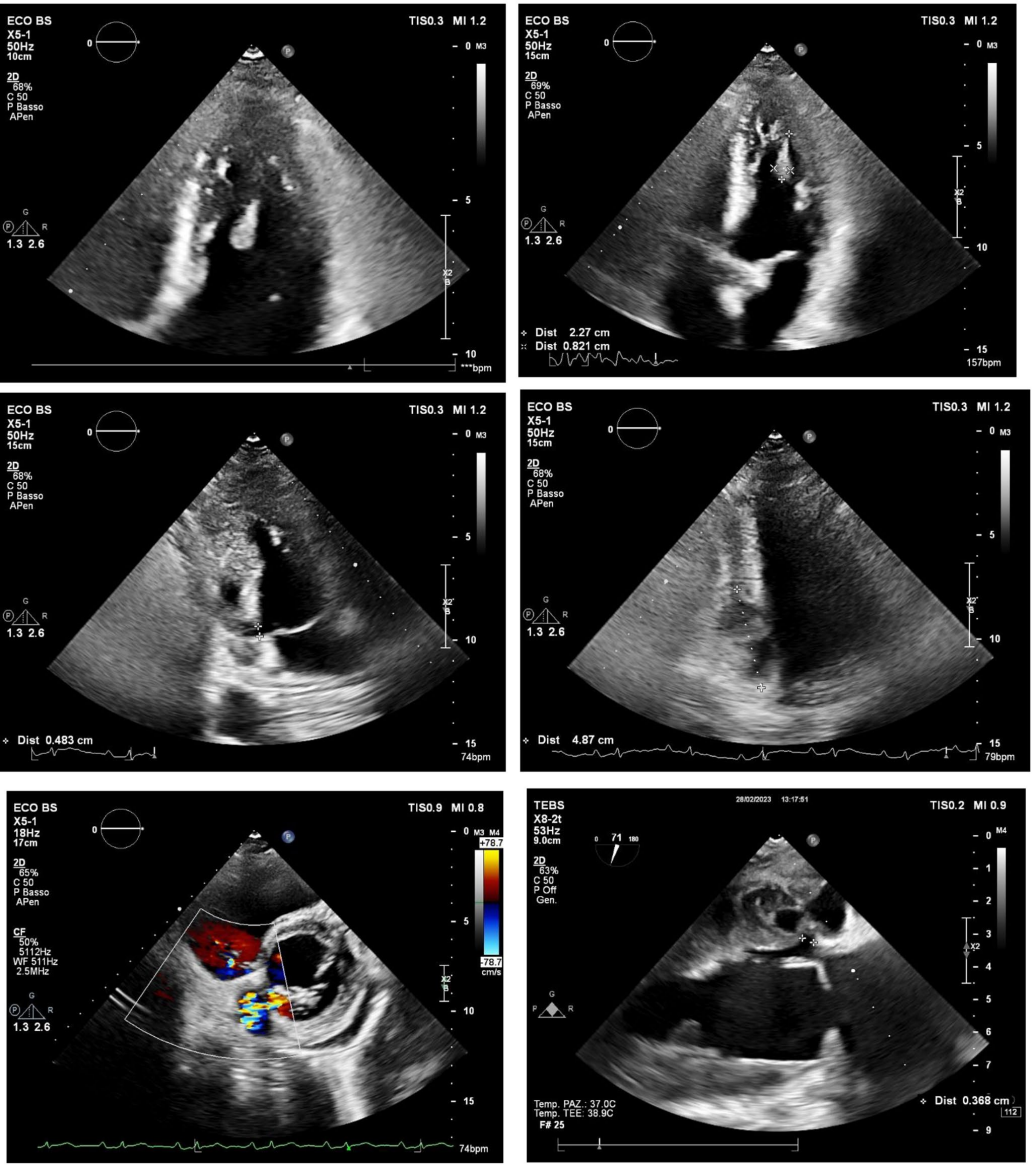
Floating pedunculate formation in the left ventricle and intramiocardial abscess seen at TTE in different echocardiographic projections

**Fig. 2:**
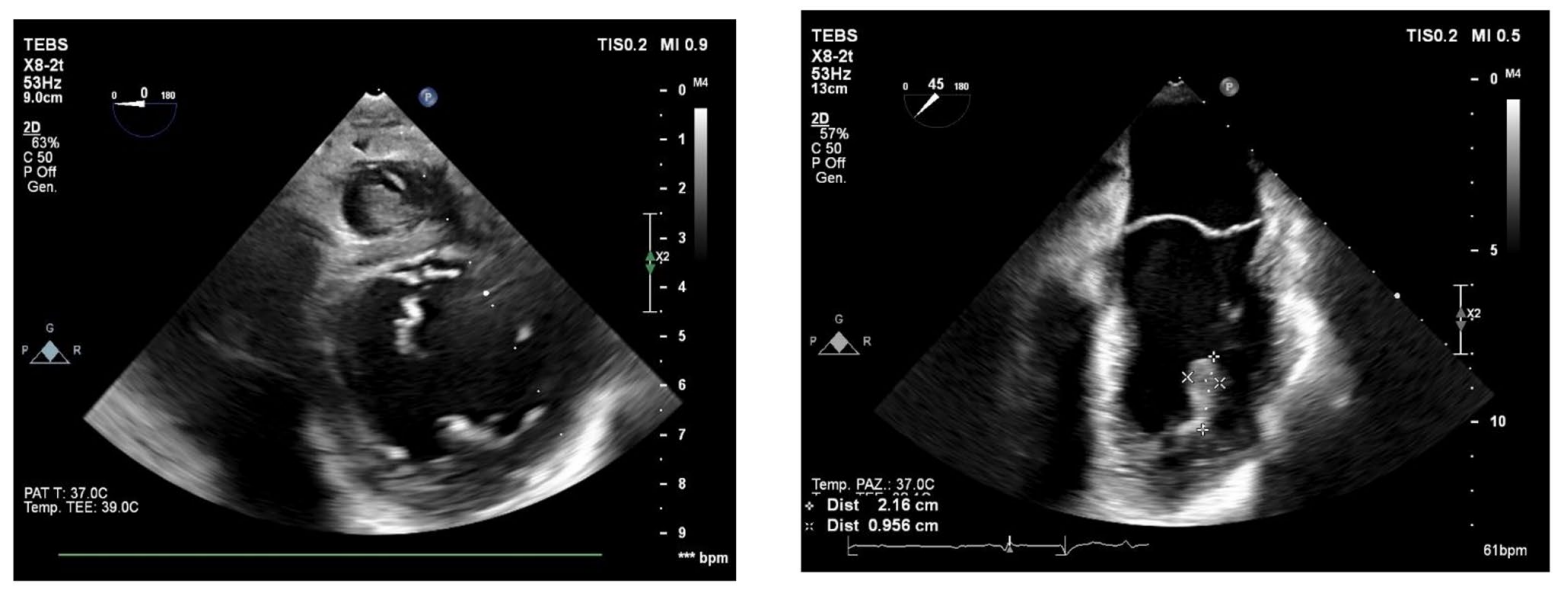
Intramyocardial abscess and endoventricular thrombosis at TEE

**Fig. 3:**
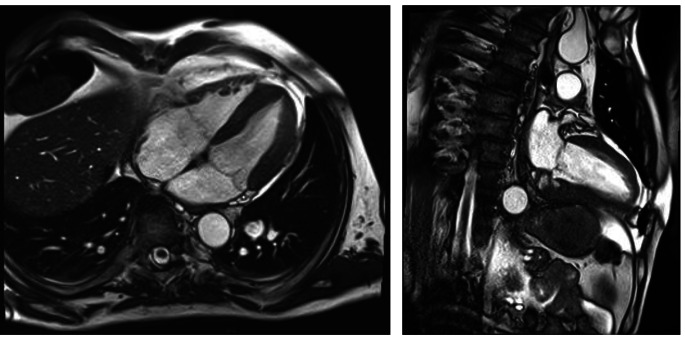
Intramyocardial abscess seen at cardiac MRI

A 57-year-old patient, without significant cardiac history, discharged about two weeks earlier from the Dermatology Unit with diagnosis of pityriasis rubra pilaris and intercurrent superficial venous thrombosis in the right lower limb (treated with enoxaparin and then replaced with antiplatelet oral therapy), has come to the Emergency Departement because of fever for a few days and a swelling on the left elbow resembling olecranon bursitis. At the clinical evaluation the patient was feverish (39 °C), suffering, and he presented erythroderma and skin desquamation. On the ECG sinus rhythm, 1st degree atioventricular block, and some diffuse alterations of ventricular repolarization in antero-lateral leads were documented. Blood tests revealed leukocytosis (WBC 12,990/uL), notable increase in inflammation indices (CRP 238 mg/l, normal value < 5 mg/l), slight increase in procalcitonin (0.54 ng/ml, n.v. < 0.5 ng/l ), TnT-hs 100  --> 100 ng/l (n.v. < 14 ng/l). Transthoracic echocardiogram (TTE) documented normal left ventricular ejection fraction (LVEF), presence of floating pedunculate formation in the left ventricle (16 × 10 mm), no significant valvular abnormalities and no inferior vena cava congestion. Infectious disease specialist gave the indication to perform blood samples, starting antibiotic therapy with Oxacillin, Ceftriaxone and Amikacin for suspected endocarditis. The patient has been recovered in Cardiology Unit for clinical and instrumental monitoring. He underwent the day after to a transesophageal echocardiography (TEE) which confirmed the presence of a shaped and peduncolated formation, attached to the anterior apex of the left ventricle, highly mobile (length 22 mm, thickness 9 mm) and small thrombi at the level of the right ventricle. Furthermore, it documented the presence of an abscess formation with multiple chambres and some internal septa, posterior to the lower left ventricular wall and extended circumferentially to the posterior mitral commissure along the posterior mitral annulus (extension approximately 5 cm), communicating with the ventricular cavity *(*Figs. 1 and 2*).* The subsequent heart MRI confirmed these findings showing the cavity extended for 4 cm at the level of the posterior-basal wall of the left ventricle, partially perfused and in communication with the atrium an ventricle, with some colliquated/thrombotic material inside *(*Fig. 3*).* Therefore intravenous unfractioned sodium heparin (UFH) infusion was started and also the antibiotic therapy was modified with Oxacillin + Daptomycin because of positive blood cultures for multisensitive Staphylococcus Aureus (MSSA). Lower limbs venous doppler ultrasound showed posterior tibial veins thrombosis on the right. Brain CT was performed to exclude any cerebral embolizations, and it was diagnostic for minute ischemic lesions in the cerebellar area and chronic ischemic gaps in the bilateral thalamic capsulo-lenticular area. During the subsequent hospitalization because of ideo-motor slowing, weakness of the lower limbs and absence of osteotendinous reflexes in the lower limbs, the Neurologist requested electromyography/electroneurography (EMG/ENG) which documented the presence of motor and sensitive polyneuropathy in the lower limbs and also MRN of cerebral vessels.

The radiological examination was diagnostic for the presence of multiple minute areas of diffusion restriction at the cerebellar hemispheric level bilaterally, occipito-polar and parietal on the right, temporo-parieto-frontal and paratrigonal on the left, attributable to recent ischemic lesions. In correspondence with the left central sulcus was described a rounded area with a maximum diameter of approximately 3 mm. This finding can’t be interpreted unequivocally and it was described as a mycotic aneurysm or an ischemic lesion with haemorrhagic infarction. Bilaterally, in T2-FLAIR sequences, there were areas of hyperintensity compatible with chronic ischemic lesions. Anticoagulant therapy with intravenous sodium heparin was confirmed by Neurologists. Fundus oculi and abdominal ultrasound were negative for embolizations. The case was also discussed in the Heart Team Meeting and the Cardiac Surgeons did not indicate an inerventional approach due to the impossibility to obtain a complete debridment of the infected myocardial site and the high surgical risk. Subsequent transthoracic echocardiograms showed a gradual disappearance of the previously described endocavitary formation.

After an initial improvement of the clinical conditions and the haemodynamic stability, we opted for switch to Warfarin. No arrythmias were registered on telemetric monitoring.

Some days later, the patient referred visual deficits and confusion, an urgent brain-CT was so performed ad it described the presence of an intraparenchymal hematoma in the right occipital temporal area (maximum diameter approximately 2.6 cm). There were no indications for emergency neurosurgical intervention, anticoagulant drugs were suspended, vitamin K and protamine sulfate were administered intravenously.

However, the patient experienced clinical worsening, with bad headache due to intracranial hypertension, requiring orotracheal intubation. Human Complex was administered and a second brain-CT was quickly performed. The exam showed a clear increase of the intraparenchymal hematoma in the temporo-parietal area with a maximum antero-posterior lenght of 9 cm, with also the presence of a right frontal subdural hematoma (8 mm thickness). The mass caused a shift on the ventricular system and on septum pellucidum of about 1.4 cm. Neurosurgeons made an emergency decompression and evacuation, and the patient was transferred to Intensive Care Unit for monitoring.

**Fig. 4:**
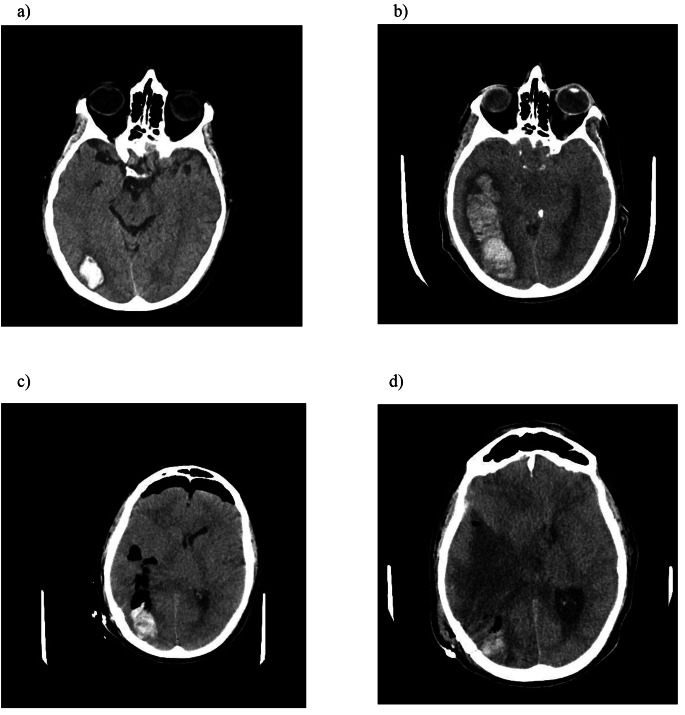
Eevolution of intraparenchymal hematoma in the temporo-parietal area: **a**) First CT performed; **b**) Second CT scan performed – hematoma maximum antero-posterior lenght 9 cm; **c**) CT after surgical decompression; **d**) Last CT performed shows only a partial reduction in the shift of the median structures with blood component and air bubbles near the surgical brain bed

In the following days the man, intubated on mechanical ventilation (GCS 3), had multiple control brain CT scans, which after the craniotomy showed only a partial reduction in the shift of the median structures, with a blood component and air bubbles near the surgical brain bed *(*Fig. 4*).* After a progressive neurological deterioration, trunk reflexes diseppeared and hemodynamic instability required norepinephrine infusion. Three days later patient brain death was declared.

## Discussion

Infective endocarditis (IE) is an important problem of public health. In 2019, an annual incidence was estimated about of 13.8 cases per 100,000 subjects, with 66,300 deaths worldwide attributable to IE. The recent ESC Endocarditis Guidelines have introduced important news especially in terms of prevention, prophylaxis and antibiotic therapy management [1]. S. Aureus and Streptococci are the most frequent etiological agents but also E. Faecalis is often reported in registries as responsible of infection.

A cardiac abscess is a suppurative leison of the myocardium, endocardium, native or prosthetic valve tissue. It occurs in the most of cases as a serious complication of an infective endocarditis (the incidence is about 30–40% in these patients and is more common in subjects with prostethic valve endocarditis - PVE) and less frequently as an isolated form caused by a dissemination related to bacteremia or a septic state. An abscess is a clear sign that an uncontrolled infection is ongoing.

Intramyocardial abscesses could also develop in cardiac areas which have previously suffered an insult, such as a recent myocardial infarction [2]. To confirm the presence of a peri-valvular involvement transesofageal echocardiogram has more sensibility and it is more specific than transthoracic echocardiogram. Cardiac CT is a useful technique to analyze the peri-valvular extension of the infection. Complications of a cardiac abscess are rhythm abnormalities on ECG such as atrioventricular blocks, in particular in patients with peri-aortic abscess because of the nearness to the atrioventricular node (AV). These subjects can also present fever, chest pain, heart failure and obviously clinical signs of embolization.

As reported in the recent Endocarditis ESC Guidelines and in the EURO-ENDO registry 11.5% of patients had conduction alterations at the diagnosis (mainly 1st, 2 nd and 3rd AVB). The new onset of AVB caused by an abscess is an indication for urgent cardiac surgery [1].

Perivalvular abscesses are also more common with prosthetic valves. In this case, the annulus instead of the leaflet is usually the primary site of infection. The degree of conduction disruption, therefore, depends on the extent of the involvement of the conduction system and is more commonly seen in perivalvular aortic abscesses. Additionally, the severe extension of perivalvular infection can also result in extrinsic coronary compression, or disruption, leading to an acute coronary syndrome. Thus far, only aortic valve involvement and current intravenous drug use have been prospectively identified as independent risk factors for a perivalvular abscess. Any patient with a cardiac abscess, regardless of all other factors, has an increased risk of embolization, morbidity, and mortality.

Khan et al. describe the case of a Proteus Mirabilis endocarditis on the aorto-tricuspidal ring in a woman with aortic bioprothesis who was undergoing hemodialysis treatment by CVC. Despite intravenous antibiotic therapy, the patient developed a perivalvular abscess that required surgical debridment [3]. Another rare case of complicated endocarditis is described by Desaint et al.: they talk about a perivalvular mitral abscess extended from an endocarditis on native mitral valve by MSSA, which became complicated with pericardial fistulization and led to septic shock with patient’s exitus [4].

Intramyocardial abscess may also occur as rare complication of myocardial infarction as a result of overinfection of the postinfarct necrotic area such as the one described by Grant et al. [5]. Less frequently in the scientific literature are described cases of Intramyocardial Abscess as a complication of cardiac surgery [6].

Our case report describes a very rare case of advanced intramyocardial abscess in communication with cardiac chambers, which occurs in a previously completely healthy heart with no evidence of infective endocarditis on the native heart valves. The patient arrived at the Emergenct Department with a left oleocranial bursitis associated to a septic state with blood cultures positive for MSSA. The bacteriemia has surely developed from the elbow infection resulting in a rooting of the pathogen in the heart muscle. The atypical dissemination of the pathogen at the level of myocardium may have been caused also by the immunosuppressive therapies that the patient had been take for months because of the treatment of Pityriasis Rubra Pilaris. The infection subsequently spread through a fistulization in the left ventricle. TTE has been the first evaluation tecnique, but TEE and Cardiac MRI gave the confirmation of diagnosis. Cardiac CT would have been surely another valid option and it is in class I of recommendation in case of suspected perivalvular complications or when TEE is not diagnostic. Surgical evaluation is crucial in these situations: large abscesses are unlikely to resolve only with antibiotic therapy and the latest ESC guidelines on Infecive Endocarditis recommend urgent surgical interventions (class I) in case of local extension of infection, such as the presence of intramyocardial abscess or fistulae [1].

Brain MRI showed the presence of multiple minute areas attributable to recent ischemic lesions. The patient was obviously evaluated by the Heart Team that judged the abscess inoperable due to the large dimensions, the elevated surgical risk and the difficult to obtain a complete debridement. He was first treated with empiric intravenous antibiotic therapy and then, after the results of the antibiograms it was switched to targeted therapy. The role of Endocarditis Team and interdisciplinary relations between cardiologist, cardiac surgeon, infectious diseases specialist are fundamental for a complete management of these patients.

The causes of thrombus formation in the left ventricle (EF was preserved) and also the presence of likely small thrombi at the level of the right ventricle have not been fully elucidated. One of the triggering factors may have been the heterozygosity mutation of coagulation factor V, some cases of this consequence have been reported in the literature [7–9]. After 5 days of anticoagulation therapy echocardiographic monitoring evidenced an almost complete resolution of the thrombotic mass and we decided for a switch to Warfarin, as reported in the recent AHA Scientific Statement on ventricular thrombosis, when patient’s conditions were stable. Some days later he developed a voluminous intraparenchymal hemorrhage with consequent endocranial hypertension. Despite the administration of vitamin K, protamine sulfate and attempted surgery, the patient subsequently died in the Intensive Care Unit.

## Data Availability

No datasets were generated or analysed during the current study.
